# Editorial: The role of validated tools, including pictorial aids, to support medication adherence and counselling

**DOI:** 10.3389/fphar.2025.1561101

**Published:** 2025-01-31

**Authors:** Piotr Merks, Agnieszka Neumann-Podczaska, Regis Vaillancourt

**Affiliations:** ^1^ Faculty of Medicine, Collegium Medicum, Cardinal Stefan Wyszyński University, Warsaw, Poland; ^2^ The Polish Pharmacy Practice Research Network (PPPRN), Warsaw, Poland; ^3^ Chair and Department of Palliative Medicine, Poznań University of Medical Sciences, Poznań, Poland; ^4^ Senior Institute, University of Economics and Human Sciences in Warsaw, Warsaw, Poland

**Keywords:** medication adherence, pictorial aids, patient safety, validation, patiem centered care

Medication adherence is a cornerstone of effective healthcare delivery, particularly for patients managing chronic diseases. However, non-adherence remains a significant global challenge, with profound implications for patient outcomes, healthcare systems, and societal well-being. As therapies become increasingly complex, innovative and validated tools are essential to support patients in understanding and adhering to their regimens (Merks et al., 2021). This Research Topic highlights the role of such tools, including pictorial aids, in enhancing medication adherence and counselling, based on insights from recent studies.

Poor medication adherence arises from various factors, including limited medication literacy—the ability to understand, interpret, and apply information about medications. Addressing medication literacy involves enhancing patients’ understanding of prescription labels, instructions, and side effects while fostering effective communication with healthcare providers (Pouliot et al., 2018). For instance, Yoon et al. explored how cultural beliefs and language barriers impact adherence among multi-ethnic Asian populations, emphasizing the importance of culturally sensitive interventions such as pictorial aids.

Validated tools are critical for tailoring interventions and ensuring consistent healthcare practices. Rusu et al. demonstrated the utility of the Hill-Bone Compliance Scale for assessing adherence in Romanian patients with cardiovascular risks (Rusu et al., 2023). Similarly, Robberechts et al. developed the BRANT-MERQS scoring table to evaluate medication reviews, identifying areas for improvement in pharmacist-physician collaboration and patient-centred care (Robberechts et al., 2023).

Visual communication through pictograms has emerged as a powerful strategy to address low health literacy and improve medication adherence. Merks et al. reviewed how pharmaceutical pictograms enhance patients’ recall and understanding of drug-related information. The effectiveness of these tools lies in their careful design, validation, and cultural adaptation. Real-world applications, such as in the work of Hu et al. and Fan et al., underscore the need for clear communication about adverse drug events, where visual aids can bridge gaps in understanding and encourage proactive management (Hu et al., 2023; Fan et al., 2023).

Dual Coding Theory provides a foundation for understanding the success of visual aids in healthcare. According to this theory, combining verbal and visual information enhances learning and memory. When integrated into patient education materials, pictograms can improve comprehension and adherence, particularly among populations with limited health literacy.

The findings in this Research Topic offer actionable insights for healthcare practice and policy. Culturally and contextually adapted tools should be integrated into clinical workflows, supported by healthcare professional training and technological innovations like interactive apps. Collaboration between pharmacists, physicians, and other healthcare providers is essential to address the multifactorial nature of non-adherence.

In conclusion, validated tools, including pictorial aids, play a pivotal role in tackling the challenge of medication adherence. This Research Topic highlights their potential to enhance communication, optimize healthcare delivery, and improve patient outcomes globally. Continued research and innovation are essential to refine these tools and ensure their effective integration into healthcare systems.
